# CD 9 and vimentin distinguish clear cell from chromophobe renal cell carcinoma

**DOI:** 10.1186/1472-6890-9-9

**Published:** 2009-11-18

**Authors:** Ariel A Williams, John PT Higgins, Hongjuan Zhao, Börje Ljunberg, James D Brooks

**Affiliations:** 1 Department of Urology, Stanford University, California, USA; 2 Department of Pathology, Stanford University, California, USA; 3 Department of Surgical and Perioperative Sciences, Urology and Andrology, Umeå University, Umeå, Sweden

## Abstract

**Background:**

Clear cell renal cell carcinoma (ccRCC) and chromophobe renal cell carcinoma (chRCC) can usually be distinguished by histologic characteristics. Occasionally, diagnosis proves challenging and diagnostic difficulty will likely increase as needle biopsies of renal lesions become more common.

**Methods:**

To identify markers that aid in differentiating ccRCC from chRCC, we used gene expression profiles to identify candidate markers that correlate with histology. 39 antisera and antibodies, including 35 for transcripts identified from gene expression profiling, were evaluated. Promising markers were tested on a tissue microarray (TMA) containing 428 renal neoplasms. Strength of staining of each core on the TMA was formally scored and the distribution of staining across different types of renal neoplasms was analyzed.

**Results:**

Based on results from initial immunohistochemical staining of multitissue titer arrays, 23 of the antisera and antibodies were selected for staining of the TMA. For 7 of these markers, strength of staining of each core on the TMA was formally scored. Vimentin (positive in ccRCC) and CD9 (positive in chRCC) best distinguished ccRCC from chRCC. The combination of vimentin negativity and CD9 positivity was found to distinguish chRCC from ccRCC with a sensitivity of 100.0% and a specificity of 95.2%.

**Conclusion:**

Based on gene expression analysis, we identify CD9 and vimentin as candidate markers for distinguishing between ccRCC and chRCC. In difficult cases and particularly when the amount of diagnostic tissue is limited, vimentin and CD9 staining could serve as a useful adjunct in the differential diagnosis of ccRCC and chRCC.

## Background

Renal cell carcinoma (RCC) is diagnosed in 55,000 patients in the United States each year, and its incidence is steadily increasing[1]. Three major histological RCC types are recognized, clear cell (conventional) RCC (ccRCC), papillary RCC (pRCC), and chromophobe RCC (chRCC)[2]. Accurate histological characterization is particularly important for risk assessment in patients who have undergone radical nephrectomy for localized disease. For patients with advanced RCC, histologic subtype is predictive of clinical outcome and of responsiveness to interleukin-2 therapy and may also affect responsiveness to tyrosine kinase inhibitors such as sunitinib and sorafanib [3-10]. Widespread use of cross-sectional imaging has led to the incidental discovery of many small renal lesions and up to 20-30% of these can be benign [11-14]. Increasingly, patients with these small lesions undergo core biopsy to document the need for treatment and as a prelude to minimally invasive treatments such as cryotherapy, radiofrequency ablation, or partial nephrectomy[11,12,14,15]. ChRCC and ccRCC demonstrate different clinical behaviors and can pose challenges in diagnosis, particularly on small tissue samples such as a core biopsy. Development of reliable diagnostic markers for these neoplasms could find application as sampling of small lesions and new targeted therapies for advanced disease expand in clinical use.

Gene expression patterns have been identified that can be used to accurately segregate the three main RCC subtypes, with ccRCC overexpressing proximal nephron, angiogenic, and immune response genes, pRCC overexpressing serine protease inhibitors and extracellular matrix genes, and chRCC overexpressing distal nephron and oxidative phosphorylation genes[16,17]. While the discoveries of genetic markers and gene expression patterns unique to RCC types have provided invaluable insight into RCC pathogenesis, genetic sequencing and gene expression profiling are currently too tedious and costly for widespread clinical use. Several immunohistochemical markers have been proposed as aids in differentiating histological subtypes of renal malignancies[18]. However, a role for additional markers still exists. Using DNA microarray analysis of a large set of tumors, we identified a set of candidate diagnostic transcripts whose levels differ significantly between ccRCC and chRCC. We evaluated protein expression of 35 candidate markers using immunohistochemistry on a tissue microarray (TMA) composed of an independent set of 249 ccRCC and 25 chRCC.

## Methods

### Gene expression profiling

Fresh frozen kidney tumor samples were obtained from Umeå University under an IRB approved protocol. Tumor histology was confirmed by 2 independent pathologists and RNA was extracted using Trizol as described previously[19]. Comprehensive transcript profiling was carried out using spotted cDNA microarrays containing 44,000 spots representing approximately 27,290 unique Unigene clusters as described. Transcript levels for the ccRCC have been reported previously and are available through Gene Expression Omnibus (GEO accession number GSE17746)[20]. Expression profiling of the ccRCC and chRCC was carried out at the same time and data from the chRCC samples are also available at GEO (accession number GSE3538).

The Significance Analysis of Microarrays (SAM) procedure was used to identify 685 transcripts differentially expressed between ccRCC and chRCC at a false discovery rate of 0.01%[21]. In this analysis, we attempted to balance the groups by using all of the 9 available chRCC and 22 of 177 ccRCC that had been randomly selected from each of the 5 ccRCC gene expression subclasses we had reported previously[19].

### Construction of RCC TMA

Archived formalin-fixed and paraffin blocked specimens of renal neoplasms with corresponding hematoxylin and eosin (H & E) stained sections were obtained from 428 radical nephrectomies performed at Stanford University between 1995 and 2006. The presence of a renal neoplasm was confirmed in each specimen after permission was obtained from the Stanford University Institutional Review Board to use these tissues for RCC TMA construction. A tissue array instrument (Beecher Instruments; Sun Prairie, WI) was used to construct the RCC TMA containing a representative core from each renal neoplasm. Cores from normal kidney, tonsil, and placenta, breast, prostate, and small cell lung carcinomas, and melanoma were included as controls. Using an H & E stained section from the RCC TMA and the original conventional slides, the diagnosis and representativeness of each core of renal tissue was confirmed. The TMA contained 249 ccRCC, 47 urothelial carcinomas, 38 pRCC, 25 chRCC, 17 oncocytomas, 13 angiomyolipomas, 3 mixed epithelial and stromal tumors, 3 mucinous tubular and spindle cell carcinomas, 3 nephroblastomas, 1 adenocortical carcinoma, 1 juxtaglomerular cell tumor, 1 mesoblastic nephroma, 1 metanephric adenoma, 1 metastatic adenocarcinoma, 1 neuroblastoma, and 3 normal kidney specimens. It also contained 13 RCCs that could not be classified, including 4 that demonstrated sarcomatoid dedifferentiation. TMA cores from 11 tumor specimens did not contain interpretable tumor tissue.

### Immunohistochemical staining

Markers were available against 35 of the differentially expressed transcripts: Custom antisera raised in rabbits to ATP-binding cassette subfamily C (ABCC) members 2 and 5, bicaudal D homolog 1 (BICD1), CD53, CD55, CD83, CD9, cyclin-dependent kinase 6 (CDK6), desmoglein 2 (DSG2), fatty acid-binding proteins (FABP) 3 and 7, soluble guanylate cyclase 1 alpha 3 (GUCY1A3), inhibitor of DNA binding 2 (ID2), interferon-induced transmembrane protein 1 (IFITM1), immunoglobulin kappa constant region (IGKC), microtubule associated serine/threonine kinase family member 4 (MAST4), MON1 homolog B (MON1B), pleckstrin homology-like domain family A member 1 (PHLDA1), periostin (POSTN), protein phosphatase 1H (PP2C domain containing) (PPM1H), S100A, solute-carrier family 16 (SLC16), succinate-CoA ligase GDP-forming beta subunit (SUCLG2), transcription factor AP2-alpha (TFAP2A), tissue factor pathway inhibitor (TFPI), transferrin receptor (TFRC), tumor necrosis factor ligand superfamily member 10 (TNFSF10), tropomyosin 1 (TPM1), trichorhinophalangeal syndrome 1 (TRPS1), and vav3 oncogene (VAV3), monoclonal antibodies to vimentin (VIM) (Dako, Glostrup Denmark), CD99 (Dako) and caldesmon (CALD) (Dako), and polyclonal antibodies to alpha-1-antitrypsin (A1AT, also known as SERPINA1) (Dako) and synuclein alpha (SNCA) (Cell Signaling Technology, Danvers MA) (Table 1). These antisera and antibodies were tested, along with custom antisera to FABP5, lymphoid-specific helicase (HELLS), and a hypothetical protein similar to RIKEN cDNA 1110014F24 gene (HYP1110014F24) and polyclonal antibody to ezrin-radixin-moesin-binding phosphoprotein (ERBP) (Abcam, Cambridge MA), which were incidentally noted to have potential as markers of chRCC in previous stainings of multi-tissue TMAs independently of the gene expression data.

**Table 1:** Staining conditions

Gene name	Clone	Source	Titer	Antigen-retrieval	Detection
ABCC2	Polyclonal	AGI	1:50	HIAR	Envision

CALD	h-CD	Dako	1:50	HIAR	Envision

CD55	Polyclonal	AGI	1:100	HIAR	Envision

CD83	Polyclonal	AGI	1:50	HIAR	Envision

CD9	Polyclonal	AGI	1:1500	HIAR	Envision

CD99	12E7	Dako	1:40	HIAR	iView

FABP3	Polyclonal	AGI	1:100	HIAR	Envision

FABP5	Polyclonal	AGI	1:200	HIAR	Envision

HELLS	Polyclonal	AGI	1:20	HIAR	Envision

HYP 1110014F24	Polyclonal	AGI	1:20	HIAR	Envision

IFITM1	Polyclonal	AGI	1:20	HIAR	Envision

IGKC	Polyclonal	AGI	1:50	HIAR	Envision

MON1B	Polyclonal	AGI	1:500	HIAR	Envision

S100A	Polyclonal	AGI	1:100	HIAR	Envision

SERPINA1	Polyclonal	Dako	1:4000	None	iView

SLC16	Polyclonal	AGI	1:200	HIAR	Envision

SUCLG2	Polyclonal	AGI	1:100	HIAR	Envision

SNCA	Polyclonal	Cell Signaling Technology	1:4000	None	Not automated

TFAP2a	Polyclonal	AGI	1:100	HIAR	Envision

TFPI	Polyclonal	AGI	1:500	HIAR	Envision

TRPS1	Polyclonal	AGI	1:200	HIAR	Envision

VAV3	Polyclonal	AGI	1:100	HIAR	Envision

VIM	Vim3B4	Dako	1:200	HIAR	Envision

Optimal titers for 34 of the markers were determined using a TMA representing multiple normal and malignant tissues (hereafter referred to as the titering TMA). We did not test antibodies to VIM, SNCA, CD99, CALD, or SERPINA1 on the titering TMA, as their performance had already been optimized and evaluated for clinical service at our institution. On the basis of initial staining of titer arrays, 16 of 34 markers were excluded from further study (Table 2). The remaining 18 of 34 markers were used, along with 5 additional antibodies, for staining of the RCC TMA. Serial 4 mm sections were used for staining with heat-induced antigen retrieval (HIAR) and signal amplification using, in all cases but one, either the Envision+ System-HRP (DAB) kit (Dako) or the iVIEW DAB Detection kit (Ventana Medical Systems, Tucson AZ). Details for all markers used to stain the RCC TMA are provided in Table 1.

**Table 2:** Characterization of staining patterns of antisera and antibodies used to stain RCC TMA

Gene name	Staining pattern
ABCC2	HB, C

CALD	C, N

CD55	AC, C

CD83	DU

CD9	M

CD99	C

FABP3	C

FABP5	HB, C

HELLS	HB, C

HYP 1110014F24	AC, C

IFITM1	DU

IGKC	HB, C

MON1B	AC, C

S100A	AC, C, M, N

SERPINA1	C, M

SLC16	DU

SUCLG2	HB, C

SNCA	NC

TFAP2a	HB, C

TFPI	HB, M

TRPS1	C

VAV3	AC, C

VIM	C, M

C = cytoplasmic, M = membranous, N = nuclear, DU = diffuse understaining, HB = high background, AC = stained all or nearly all cores, NC = stained no or very few cores

Stained sections of the RCC TMA were scored as 0 (tissue present but no staining observed), 1 (no relevant tissue present or equivocal/uninterpretable staining), 2 (weak staining), or 3 (strong staining). Strong staining was defined as staining of high intensity in more than 50% of the neoplastic cells. Weak staining represented either diffuse staining of low intensity or high intensity staining in fewer than 50% of the neoplastic cells. Formal scoring was not performed if, on inspection, the marker stained all, none, or a marginal number of cores or if a clear threshold was not apparent for positive versus negative staining (Table 2). Staining data for each marker was linked to a master data sheet containing the identities of the tissue represented in each core of the TMA. Staining data from multiple antisera was organized and manipulated using TMA-Deconvoluter software that allows organization of staining data into a single spreadsheet[22]. This transformation of the data allowed ready assessment of the degree and pattern of staining of multiple antisera across several tissue types.

## Results

### Gene expression profiling

**Figure 1:**
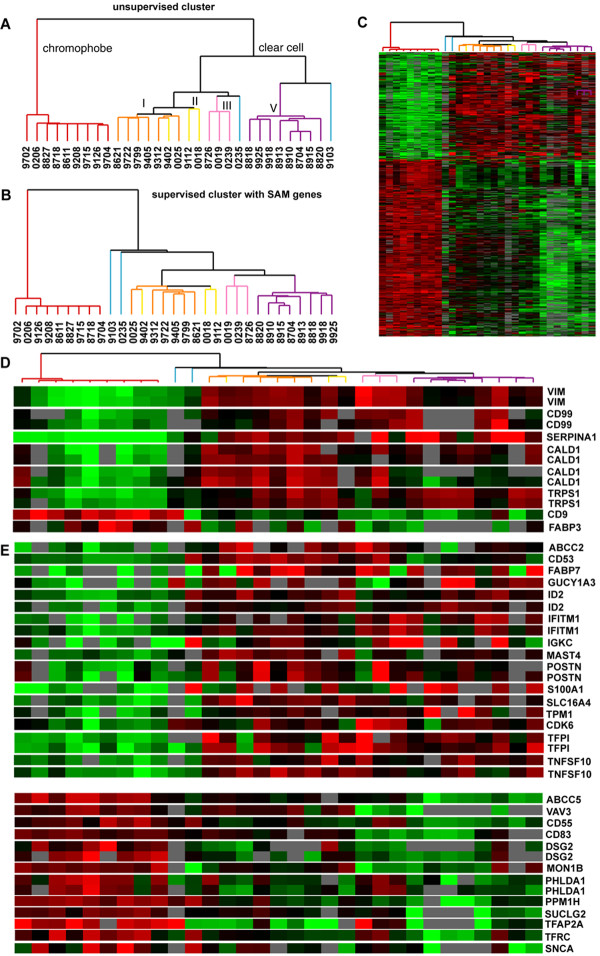
**Gene expression profiling of 22 ccRCC and 9 chRCC**. (A) Dendrogram of unsupervised hierarchical cluster analysis of the 31 samples with chRCC in red and subtypes I, II, III, IV, and V of ccRCC identified previously in orange, yellow, pink, blue and purple, respectively. (B) Dendrogram of supervised cluster analysis with list of genes identified by SAM procedure as being differentially expressed between ccRCC and chRCC. (C) Image of supervised cluster analysis with each row representing a single gene and each column a patient sample. The degree of color saturation corresponds to the ratio of gene expression in each sample compared to the mean expression across all samples. (D) Genes whose expression levels were determined using RCC TMA and for which scoring was performed. (E) Genes whose expression levels were determined using titering TMA or RCC TMA and for which scoring was not performed.

Gene expression profiling was performed on 9 chRCC removed between 1986 and 2002. We compared the transcript levels of these tumors to 22 ccRCC analyzed at the same time and on the same platform[19]. We selected genes that were well measured (2.5-fold fluorescence intensity over background) in at least 70% of the experiments and varied by 4-fold across at least 3 experiments, resulting in 3010 transcripts. Unsupervised hierarchical cluster analysis demonstrated that the chRCC showed distinct gene expression features from the ccRCC (Figure 1A). To identify transcripts that were significantly different between chRCC and ccRCC, we used the Significance Analysis of Microarrays (SAM) procedure using the 3010 transcripts. We identified 1045 transcripts representing 685 unique named genes that were significantly different between chRCC and ccRCC with a false detection rate of 0.01%. Clustering using this gene list showed a high degree of separation between the two tumor types (Figure 1B and 1C).

### Immunohistochemical staining

From the SAM list of 685 genes, we selected 35 for which antisera or commercial antibodies were available (Figures 1D and 1E). We also selected 4 antisera and antibodies that we considered promising based on previous staining of multi-tissue TMAs independently of the gene expression data. Since many of them had not been evaluated previously, we optimized the staining of all antisera and the antibody to ERBP on a titering TMA. Of the 34 markers initially tested on the titering TMA, 16 were not pursued further for a variety of reasons, including high background staining, diffuse understaining, and failure to differentiate between different tissue types (Table 2). The remaining 18 antisera and 5 antibodies were used for staining of a RCC TMA with 18 different tumor types. The RCC TMA was stained for: ABCC2, CALD, CD55, CD83, CD9, CD99, FABP3, FABP5, HELLS, HYP1110014F24, IFITM1, IGKC, MON1B, S100A, SERPINA1, SLC16, SUCLG2, SNCA, TFAP2A, TFPI, TRPS1, VAV3 and VIM (Table 2). Of these 23 markers, 7 (CALD, CD9, CD99, FABP3, SERPINA1, TRPS1, and VIM) produced differential and promising staining of the RCC TMA and were formally scored. The relative transcript levels of these genes across all samples are shown in Figure 1D. The distribution of staining across ccRCC, chRCC, pRCC, urothelial carcinomas, oncocytomas, and angiomyolipomas is shown in Table 3.

**Table 3:** Staining results of evaluable cores (%)

		CALD	CD9	CD99	FABP3	SERPINA1	TRPS1	VIM
**Clear cell RCC**	No stain	235/235 (100.0)	215/229 (93.9)	187/213 (87.8)	137/232 (59.1)	105/217 (48.4)	208/212 (98.1)	134/220 (60.9)
	Weak	0/235 (0.0)	10/229 (4.4)	21/213 (9.9)	53/232 (22.8)	63/217 (29.0)	2/212 (0.9)	28/220 (12.7)
	Strong	0/235 (0.0)	4/229 (1.7)	5/213 (2.3)	42/232 (18.1)	49/217 (22.6)	2/212 (0.9)	58/220 (26.4)

**Chomophobe RCC**	No stain	24/24 (100.0)	0/24 (0.0)	13/23 (56.5)	13/24 (54.2)	1/22 (4.5)	6/22 (27.3)	24/24 (100.0)
	Weak	0/24 (0.0)	2/24 (8.3)	7/23 (30.4)	5/24 (20.8)	7/22 (31.8)	11/22 (50.0)	0/24 (0.0)
	Strong	0/24 (0.0)	22/24 (91.7)	3/23 (13.0)	6/24 (25.0)	14/22 (63.6)	5/22 (22.7)	0/24 (0.0)

**Papillary RCC**	No stain	37/37 (100.0)	36/36 (100.0)	20/32 (62.5)	17/36 (47.2)	20/34 (58.8)	31/31 (100.0)	16/34 (47.1)
	Weak	0/37 (0.0)	0/36 (0.0)	7/32 (21.9)	9/36 (25.0)	10/34 (29.4)	0/31 (0.0)	10/34 (29.4)
	Strong	0/37 (0.0)	0/36 (0.0)	5/32 (15.6)	10/36 (27.8)	4/34 (11.8)	0/31 (0.0)	8/34 (23.5)

**Unclassified RCC**	No stain	13/13 (100.0)	10/13 (76.9)	4/9 (44.4)	7/13 (53.8)	6/11 (54.5)	10/11 (90.9)	4/12 (33.3)
	Weak	0/13 (0.0)	1/13 (7.7)	3/9 (33.3)	3/13 (23.1)	4/11 (36.4)	1/11 (9.1)	2/12 (16.7)
	Strong	0/13 (0.0)	2/13 (15.4)	2/9 (22.2)	3/13 (23.1)	1/11 (9.1)	0/11 (0.0)	6/12 (50.0)

**Urothelial carcinoma**	No stain	45/45 (100.0)	46/46 (100.0)	38/43 (88.4)	29/46 (63.0)	23/47 (48.9)	38/44 (86.4)	40/43 (93.0)
	Weak	0/45 (0.0)	0/46 (0.0)	4/43 (9.3)	12/46 (26.1)	19/47 (40.4)	5/44 (11.4)	1/43 (2.3)
	Strong	0/45 (0.0)	0/46 (0.0)	1/43 (2.3)	5/46 (10.9)	5/47 (10.6)	1/44 (2.3)	2/43 (4.7)

**Oncocytoma**	No stain	16/16 (100.0)	7/16 (43.8)	1/13 (7.7)	3/16 (18.75)	13/16 (81.3)	9/15 (60.0)	16/16 (100.0)
	Weak	0/16 (0.0)	6/16 (37.5)	2/13 (15.4)	6/16 (37.5)	2/16 (12.5)	4/15 (26.7)	0/16 (0.0)
	Strong	0/16 (0.0)	3/16 (18.8)	10/13 (76.9)	7/16 (43.8)	1/16 (6.3)	2/15 (13.3)	0/16 (0.0)

**Angiomyolipoma**	No stain	1/11 (9.1)	10/10 (100.0)	5/10 (50.0)	7/10 (70.0)	3/11 (27.3)	7/12 (58.3)	6/8 (75.0)
	Weak	0/11 (0.0)	0/10 (0.0)	5/10 (50.0)	3/10 (30.0)	5/11 (45.5)	5/12 (41.7)	1/8 (12.5)
	Strong	10/11 (90.9)	0/10 (0.0)	0/10 (0.0)	0/10 (0.0)	3/11 (27.3)	0/12 (0.0)	1/8 (12.5)

**Figure 2:**
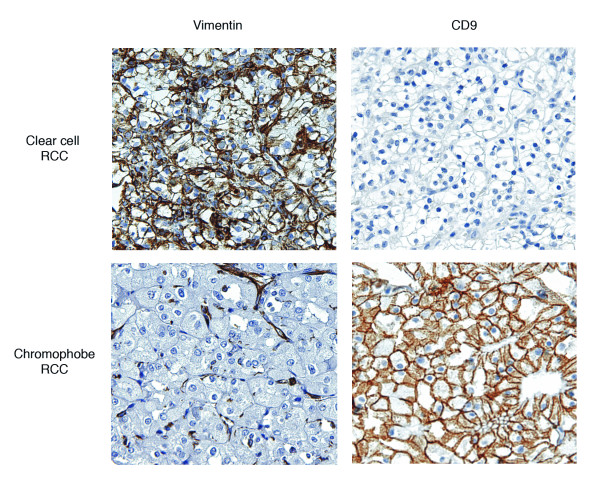
**Representative ccRCC and chRCC cores on RCC TMA stained for vimentin and CD9**.

Of all the potential subtype markers assessed, staining for CD9 and vimentin appeared to best distinguish between ccRCC and chRCC (representative staining is shown in Figure 2). 22 chRCC showed strong CD9 expression and 2 showed weak expression out of a total of 24 whereas 4 ccRCC showed strong CD9 expression and 10 weak expression out of a total of 229 (p < 0.0001 for weak or strong positive staining in ccRCC versus chRCC by Fisher exact test). Strong CD9 positivity had 91.7% sensitivity and 98.3% specificity for chRCC as opposed to ccRCC. Weak *or *strong CD9 positivity had 100.0% sensitivity and 93.9% specificity. No chRCC showed weak or strong vimentin expression out of a total of 24 whereas 58 ccRCC showed strong vimentin expression and 28 weak expression out of a total of 220 (p = 0.0003 for weak or strong positive staining in ccRCC versus chRCC by χ^2 ^analysis w/continuity correction). Although no chRCC stained for vimentin, the lack of vimentin expression was not specific for chRCC since 60.9% of ccRCC also failed to express vimentin. Combining the two stains such that only CD9 positive and vimentin negative samples were identified as chRCC improved predictive value only marginally, yielding 100.0% sensitivity and 95.2% specificity.

Besides chRCC and a small minority of ccRCC, subsets of unclassified RCC and oncocytomas stained positive for CD9. 1 unclassified RCC was weakly positive for CD9 and 2 unclassified RCC were strongly positive for CD9 out of a total of 13. 2 unclassified RCC were weakly positive for vimentin and 6 were strongly positive for vimentin out of a total of 12. No unclassified RCC were positive for both CD9 and vimentin. 6 oncocytomas were weakly positive for CD9 and 3 oncocytomas were strongly positive for CD9 out of a total of 19. None of the oncocytomas stained for vimentin.

## Discussion

Distinction of ccRCC from chRCC is typically straightforward; however, challenging cases exist and these two RCC subtypes with radically different genetic changes have been confused in clinical practice. We and others have found Hale's colloidal iron staining to be a complicated and unreliable method for identifying chRCC [23-28]. This diagnostic difficulty can only be expected to increase as the use of core needle biopsy for the diagnosis of renal neoplasms becomes more popular. Furthermore, the clinical relevance of the distinction can only be expected to increase. Several targeted therapies for RCC appear to be more effective in ccRCC than in chRCC[5,7-10]. In addition, patients may be selected for observation if they have chRCC and are at high risk for surgery[11,29,30]. Therefore, there are clear clinical needs for sensitive and specific immunohistochemical markers of ccRCC and chRCC.

We have identified candidate markers for ccRCC and chRCC using cDNA microarrays. The most promising of these have been further evaluated by immunohistochemistry on a TMA containing 428 renal neoplasms, including 249 ccRCC and 25 chRCC. Our finding of relatively poor performance of antisera raised against proteins encoded by transcripts identified by gene expression profiling is somewhat disappointing but not surprising. Many factors, including low expression of cognate proteins, poor binding affinity or specificity of the antisera, and high background signal and other technical factors can complicate immunohistochemical staining performance. In addition, post-translational regulation of proteins can lead to poor correlation between transcript and protein levels. We have encountered poor correlation of protein expression by IHC with mRNA expression by cDNA array in other disease contexts.

Despite these their limitations, transcript profiles can yield novel diagnostic and prognostic markers in a variety of diseases [31-33]. Therefore, the diagnostic utility of the transcripts we identified as differentially expressed between ccRCC and chRCC could be evaluated as reagents suitable for immunohistochemistry become available. Testing these immunohistochemical reagents on diverse tumors types can lead to potentially interesting serendipitous findings with little extra labor. For instance, despite showing elevated transcript levels in ccRCC, caldesmon showed little expression in any of the epithelial cancers but was highly specific for angiomyolipoma. Further evaluation of caldesmon as a marker for angiomyolipomas is warranted.

Vimentin and CD9 emerged as the best stains to allow distinction between the RCC types and perform at levels that suggest they can be used for clinical diagnosis. On these grounds, strong but not weak CD9 positivity could be used to identify chRCC. It is possible that the addition of vimentin staining in cases with ambiguous staining results for CD9 could improve diagnostic accuracy, although additional testing on larger sets of samples will be necessary to test this hypothesis.

Vimentin has been reported previously as a specific marker for ccRCC [34-38]. However, the relatively low frequency of positive vimentin staining in the ccRCC included in our study suggests that vimentin by itself is not an optimal biomarker of ccRCC. On the other hand, positive staining for CD9 appears to better distinguish between ccRCC and chRCC. CD9 staining of chRCC has been reported previously in a study of 66 ccRCC and 5 chRCC; however, in that study CD9 was thought to be a highly sensitive but relatively nonspecific marker of chRCC and pRCC[39]. Based on more extensive testing in a larger series of tumors, our data suggest that CD9 staining has strong potential for use as a clinically useful and highly specific marker for chRCC.

CD9 is a member of the tetraspanin family of proteins, which function via their associations with other tetraspanins, integrins, and other proteins to form what has been termed the 'tetraspanin web' [40]. CD9 is expressed at high levels in the collecting ducts, loops of Henle, and distal tubules of the normal kidney, where tetraspanins help maintain glomerular architecture and may act as important regulators of vesicular trafficking and apical sorting[39,41]. CD9 in particular has been found to be upregulated in response to osmotic stress in two renal epithelial cell lines, suggesting that it may play a role in modulating cellular adaptation to hypertonicity[42].

CD9 downregulation is a poor prognostic factor in many cancers, with CD9 expression inversely correlating with invasiveness in melanoma, breast carcinoma, squamous cell carcinoma, ovarian cancer, and cervical carcinoma [43-52]. There are multiple mechanisms by which it appears to exert its action as a metastasis suppressor[53]. CD9 may decrease transformation and cellular motility by suppressing Wnt signaling pathways and altering the expression of integrins and other adhesion molecules[54,55]. CD9 has also been proposed to regulate apoptosis via the TGF α and EGFR signaling pathways and to interfere with cell migration via the downregulation of WAVE2, an inducer of actin polymerization [56-58]. Finally, CD9 has been shown to interfere with Aggrus-mediated platelet aggregation, which may prevent tumor spread by subjecting metastatic cells to a less protective environment[59,60].

Nakamoto et al found that administration of anti-human CD9 antibody to mice inoculated with a human gastric cancer cell line resulted in reduced tumor volume, increased apoptotic indexes, and decreased tumor microvessel density, suggesting that CD9 may indeed have potential as a therapeutic target[61]. Whether CD9 expression has the same favorable prognostic value in RCC as it does in other tumor types has not been established. However, its marked downregulation in ccRCC and ppRCC may contribute to the poor prognosis of these RCC subtypes and investigation of the effects of in vivo modulation of CD9 signaling in RCC could be productive.

Like ccRCC and chRCC, oncocytoma and chRCC can be difficult to differentiate based on H & E slides alone. Potential immunohistochemical markers have been identified using small sets of tumors, but results have not been consistent[62,63]. None of the markers tested in this study were sensitive and specific for oncocytoma. CD9 staining could be marginally helpful in distinguishing between chRCC and oncocytoma, as CD9 negativity was highly specific for oncocytoma as compared to chRCC. However, as more than half of oncocytomas were CD9 positive, the routine use of CD9 to differentiate these two tumor types would be low yield. Our failure to identify a good marker of oncocytoma is not surprising, as marker selection was based on gene expression differences between ccRCC and chRCC only and the genetic profiles of oncocytoma and chRCC appear to be remarkably similar[64]. However, some gene expression differences between chRCC and oncocytoma have been recently identified and could be a valuable focus of future study[65].

## Conclusion

In summary, based on gene expression analysis, we identify CD9 and vimentin as candidate markers for distinguishing between ccRCC and chRCC. Based on the higher rate of differential staining between the RCC types, CD9 appears to be a superior marker. While the addition of vimentin to CD9 staining slightly improved discrimination, we doubt that both markers need to be used routinely in the clinical setting. With the increasing use of active surveillance, minimally invasive procedures such as cryotherapy and biologic therapies specific for cancers of clear cell histology, accurate histological diagnosis, particularly on small samples such as core biopsies, will have important therapeutic implications.

## Competing interests

The authors declare that they have no competing interests.

## Authors' contributions

AAW was responsible for acquisition of samples, design of the TMA, all immunohistochemical stains, interpretation of the stains, and data analysis and was primarily responsible for the drafting of the manuscript. JPTH co-conceived of the study and participated in its design. He was responsible for sample acquisition, design and creation of the TMA, and quality control and interpretation of immunohistochemical stains. He contributed to data analysis and contributed substantially to all phases of manuscript preparation. HZ was responsible for the gene expression analysis of the kidney tumors and subsequent analysis of the gene expression data. She also contributed to drafting of the manuscript. BL co-conceived the study and participated in its design. He provided the fresh frozen kidney tumors for gene expression analysis. He contributed to drafting of the manuscript. JDB co-conceived the study and was primarily responsible for its design. He participated in analysis of gene expression data, supervised immunohistochemistry, and coordinated all aspects of the study. He contributed substantially to all stages of preparation of the manuscript. All authors read and approved the final manuscript.

## Pre-publication history

The pre-publication history for this paper can be accessed here:

http://www.biomedcentral.com/1472-6890/9/9/prepub
